# Supplementary material for: Mid-Holocene pulse of thinning in the Weddell Sea sector of the West Antarctic ice sheet
Source: Nat Commun. 2016 Aug 22;7:12511. doi: 10.1038/ncomms12511 (PMC4996935; doi:10.1038/ncomms12511)
Supplement: Supplementary Information — Supplementary Figures 1-4 and Supplementary References [file ncomms12511-s1.pdf]

Supplementary Information:

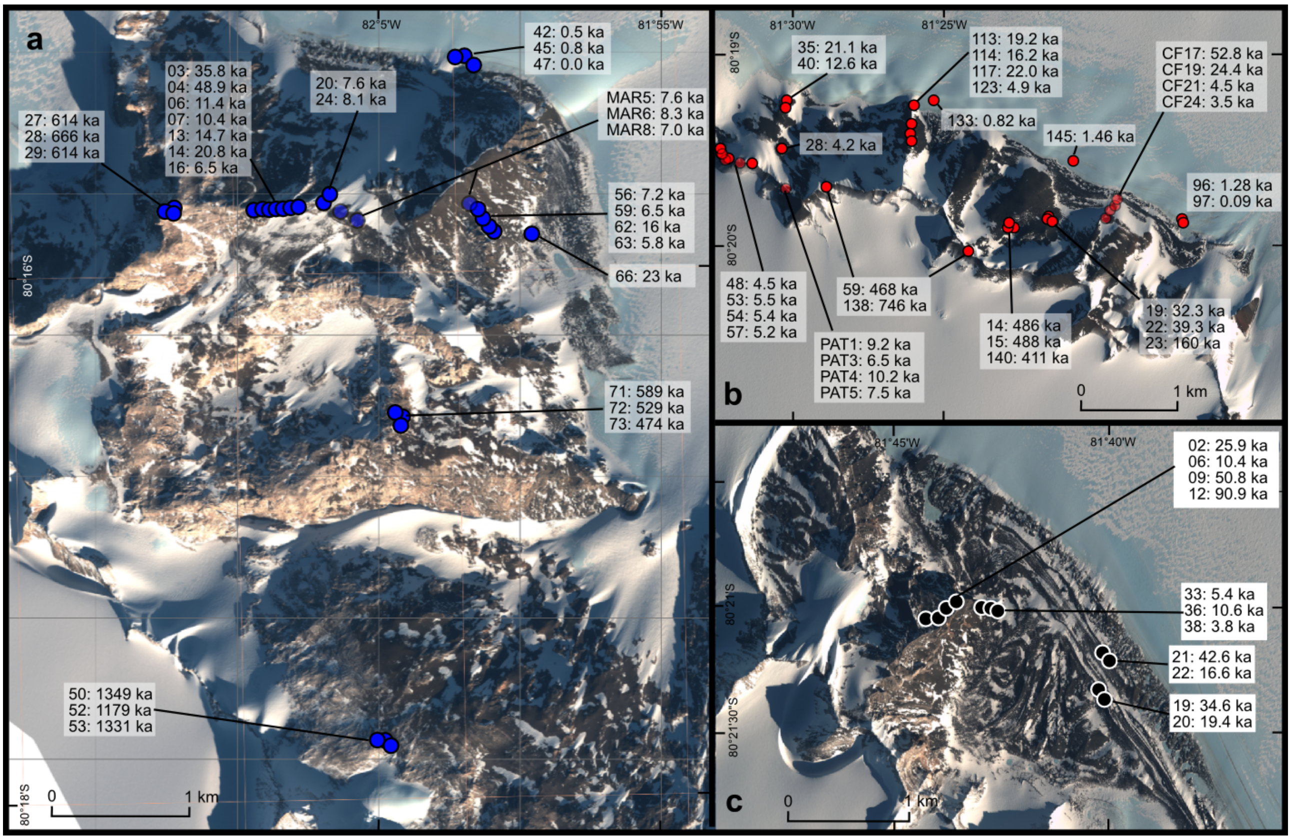

Supplementary Figure 1. Sample locations and exposure ages. The figure displays Quickbird imagery of the (a) Marble Hills, (b) Patriot Hills and (c) Independence Hills showing the sample locations and ^{10}Be exposure ages; the sample IDs are prefixed with MH12, PH12 and IH12, respectively. The light-coloured symbols with different sample IDs are from an earlier study[1]. The colours are consistent with those in Supplementary Figure 3.

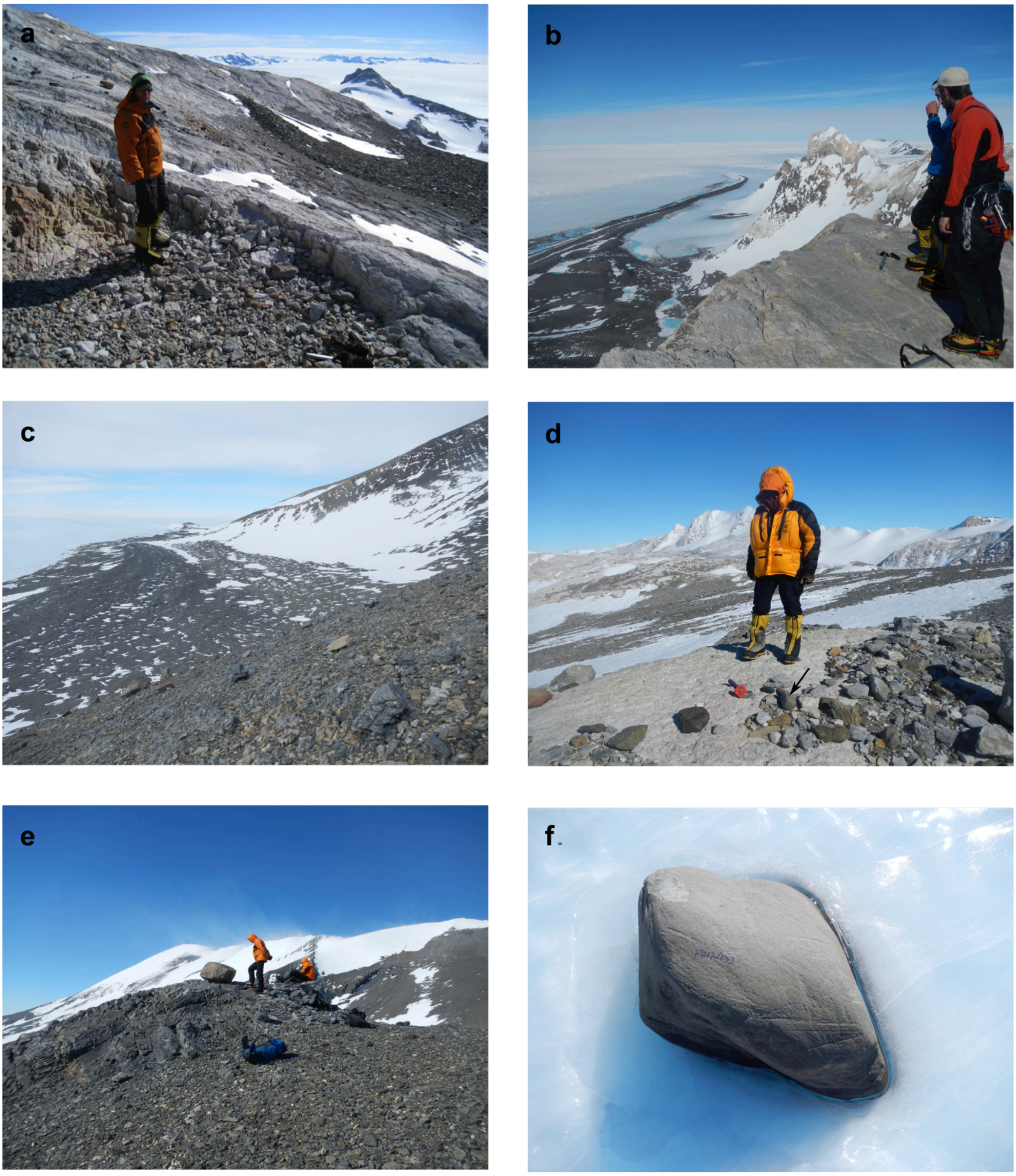

Supplementary Figure 2. Photographs of key geomorphologic features and rock samples. (a) A moraine ridge marking the upper limit of little-weathered tills in the Marble Hills. (b) The elongated medial blue-ice moraine fronting the Independence Hills demonstrates eastward ice flow in the past. (c) The blue-ice moraine in the Patriot Hills. Clasts are exposed on this supraglacial moraine before being deposited on the mountain flank. (d) The clast (arrow) near the upper limit in the Marble Hills marking the onset of thinning with an age of

6.5±0.3 ka (MH12-16). The thin drift over bedrock is typical for this area. (e) A boulder isolated on a bedrock bump in the Patriot Hills with an age of 4.2±0.4 ka (PH12-28). (f) A striated cobble emerging from the ice in front of the Patriot Hills with an age of 0.8±0.2 ka (PH12-133).

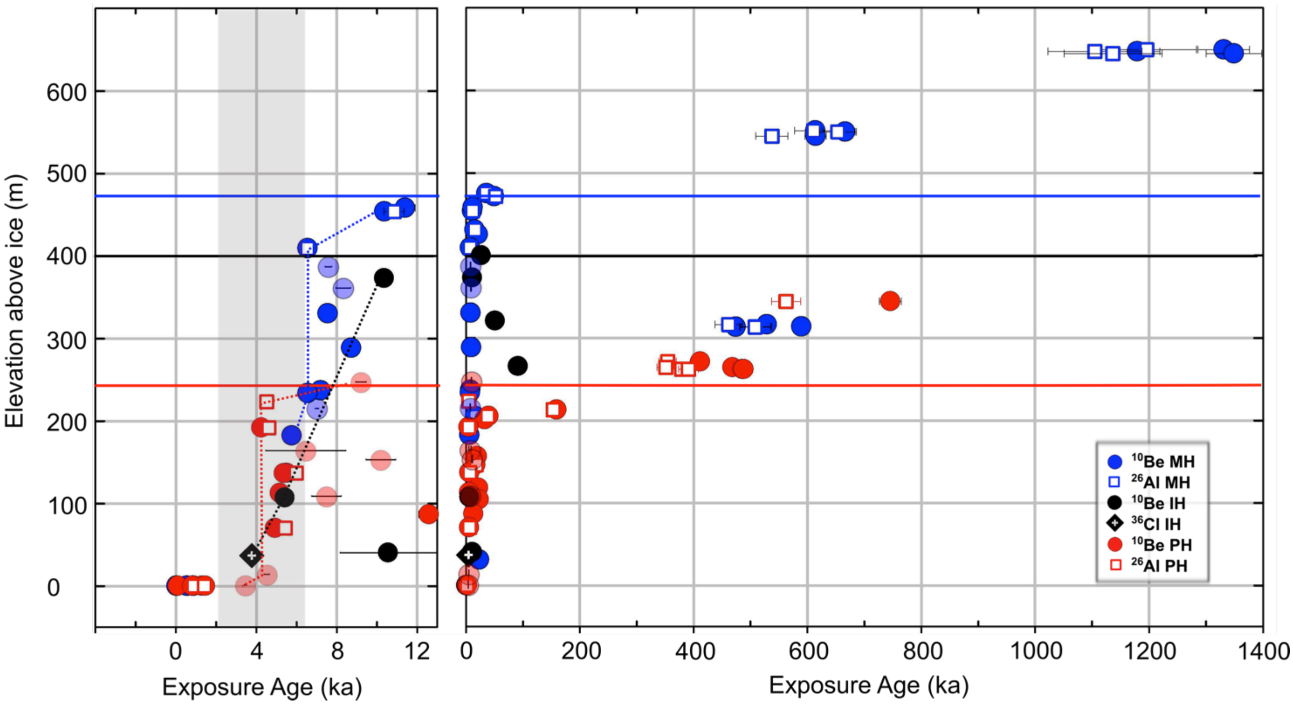

Supplementary Figure 3. The apparent exposure ages vs. elevation above the present ice surface. The figure shows results for the Marble Hills (MH), Independence Hills (IH) and Patriot Hills (PH) for the Holocene period (left panel) and the past 1.4 Ma (right panel). Error bars (1σ) reflect analytical uncertainties only. The light-coloured symbols are previously published data[1]. The solid horizontal line is the upper limit of little-weathered erratics for the MH (blue), IH (black) and PH (red). The dotted lines connect the youngest exposure dates at each massif, which are used to infer the mid-Holocene pulse of thinning as indicated by the grey shaded box. Samples located above the weathering break are significantly older[2].

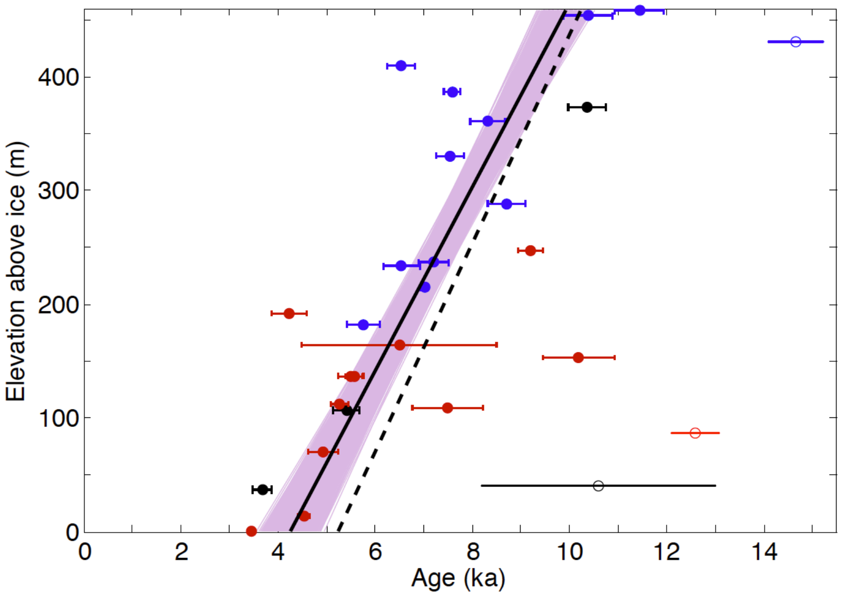

Supplementary Figure 4. Thinning-rate modelling results when all deglacial exposure ages are considered. In this model we include all exposure ages <15 ka in the linear regression to determine the modelled timing for the onset of initial thinning in the region. This contrasts with Figure 5 where we only fit exposure ages (<10 ka) that span the inferred pulse of thinning. Thinning rates are produced using 10,000 iterations of linear regression through the ^{10}Be data points and their uncertainties. For each iteration, the age for each point is randomly chosen from within the uncertainty bounds on that sample and then a linear regression is fit to those points. Uncertainties (1σ) are calculated statistically so that 68% of the resulting slope values fall within the given range. The data points are coloured to match the different massifs with blue from Marble Hills, red from Patriot Hills and black from Independence Hills. The regression excludes three samples (open circles), MH12-13 (14.7 ka), PH12-40 (12.6 ka) and IH12-36 (10.6 ka), because they are clear outliers (3σ) when compared to other exposure ages at similar elevations. The black dashed line indicates the regression that includes the outliers, but this regression fails to fit the mid-Holocene exposure ages well. The modelling suggests the onset of initial deglaciation was at 9-10.5 ka, with a lower average thinning rate of 8.1±0.2 cm a^{-1}. This result is similar to the onset of deglaciation indicated using the youngest exposure age results at c. 10 ka.

Supplementary References

1 Bentley, M. J. *et al.* Deglacial history of the West Antarctic Ice Sheet in the Weddell Sea embayment: Constraints on past ice volume change. *Geology* **38**, 411-414, doi:10.1130/g30754.1 (2010).

2 Hein, A. S. *et al.* Evidence for the stability of the West Antarctic Ice Sheet divide for 1.4 million years. *Nat Commun* **7**, doi:10.1038/ncomms10325 (2016).
